# Case Report: Alectinib and CNS-directed therapy for primary or relapsed ALK-positive anaplastic large cell lymphoma with central nervous system involvement

**DOI:** 10.3389/fonc.2026.1679620

**Published:** 2026-06-09

**Authors:** Milda Mikalonytė, Vilhelmas Landsbergis, Lukas Kevličius, Skirmantė Černauskienė, Linas Davainis, Andrius Žučenka, Igoris Trociukas, Regina Pileckytė, Birutė Davainienė, Inga Šlepikienė, Gintarė Ražanskienė, Ugnius Mickys, Jonas Ražanskas, Jūratė Dementavičienė, Mindaugas Stoškus, Raminta Batiuškaitė, Laimonas Griškevičius

**Affiliations:** 1 Hematology, Oncology and Transfusion Medicine Centre, Vilnius University Hospital Santaros Klinikos, Vilnius, Lithuania; 2 Hematology and Oncology Department, Faculty of Medicine, Institute of Clinical Medicine, Vilnius University, Vilnius, Lithuania; 3 National Centre of Pathology, Affiliate of Vilnius University Hospital Santaros Klinikos, Vilnius, Lithuania

**Keywords:** alectinib, ALK+, ALK-positive ALCL, anaplastic large cell lymphoma, CNS

## Abstract

ALK-positive anaplastic large cell lymphoma (ALCL) is a rare T-cell lymphoma, typically associated with a favorable prognosis. However, outcomes in refractory or relapsed ALK-positive ALCL with central nervous system (CNS) involvement are poor, and no standard therapy has been established. Emerging data suggest that ALK inhibitors may be safe and effective for CNS ALK-positive ALCL. Here, we present two cases of ALK-positive ALCL with CNS involvement. The first patient, an 18-year-old male was diagnosed with ALK-positive ALCL and achieved complete remission after six cycles of chemotherapy and autologous hematopoietic stem cell transplantation (HSCT) but subsequently relapsed in the CNS. He received intrathecal chemotherapy and alectinib, followed by consolidation with allogeneic HSCT, and has remained in remission for over 81 months on alectinib maintenance. The second patient, a 31-year-old woman, presented with symptoms of meningitis and was diagnosed with ALK-positive ALCL with primary isolated CNS involvement. She received alectinib combined with CNS-directed chemotherapy and has remained in complete remission for 20 months on alectinib maintenance. These cases suggest that alectinib, combined with CNS-directed chemotherapy, is effective in both ALK-positive ALCL with CNS relapse and primary isolated CNS disease.

## Introduction

Anaplastic large cell lymphoma (ALCL) is a rare peripheral T-cell lymphoma (PTCL), accounting for approximately 2–3% of all adult non-Hodgkin lymphoma cases (1). In adults, approximately 40-50% of ALCLs constitutively express anaplastic lymphoma kinase (ALK) protein, encoded by oncogenic fusions involving the *ALK* gene (2–5). ALK-positive ALCL predominantly affects young male patients and is generally associated with a favorable prognosis, with a 5-year overall survival rate of 81% (4, 6, 7).

Central nervous system (CNS) involvement, either as relapse or primary disease, is infrequent, occurring in approximately 2–6% of cases, and is associated with a dismal prognosis, with reported survival of 1–7 months (8, 9). No standard therapy has been established for patients with CNS involvement, and reported treatment approaches vary considerably. Recent data suggest promising efficacy of second-generation ALK inhibitors, such as alectinib, in relapsed or refractory ALK-positive ALCL with CNS involvement (10–14).

We present two cases of ALK-positive ALCL with CNS involvement, one with systemic relapse and one with primary isolated CNS disease, both successfully treated with alectinib.

## Case description

### Case 1

An 18-year-old previously healthy male presented with an enlarged axillary lymph node in November 2017. Lymph node biopsy confirmed ALK-positive, CD30-positive ALCL harboring the t(2;5)(p23;q35) *NPM1::ALK* fusion gene (**Supplementary Figure 1**). PET/CT scan showed metabolically active lymph nodes above and below the diaphragm, consistent with stage III disease. The patient received six cycles of CHOEP chemotherapy, achieving a PET-negative complete response (CR), and subsequently underwent consolidation with BEAM-conditioned autologous hematopoietic stem cell transplantation (HSCT) in March 2018. Two months after transplantation, he presented with fever and lymphadenopathy. Relapse of ALK-positive ALCL was confirmed on PET/CT, which demonstrated generalized lymph node involvement with multiple lesions in bones, lungs, liver, spleen, pancreas, stomach, and small intestine (**Figure 1A**). The disease progressed despite salvage therapy with one course of brentuximab vedotin (BV) and HD-MTX–based non-Hodgkin lymphoma-Berlin-Frankfurt-Münster (NHL-BFM) regimens. Subsequently, treatment with crizotinib (250 mg twice daily) was initiated, and the patient’s condition improved. After two weeks of treatment with crizotinib, the patient was re-admitted with generalized seizures. Brain MRI (**Figures 1D, E**) and detection of the *NPM1::ALK* fusion transcript in CSF confirmed CNS progression, although multiparameter flow cytometry did not reveal an immunophenotypically aberrant population, systemic progression was not evaluated at the time. Given the lack of CNS disease control with crizotinib, treatment was discontinued and alectinib (600 mg twice daily) was initiated alongside three doses of intrathecal triplet therapy (cytarabine, methotrexate, dexamethasone). Three weeks after initiating therapy, complete CNS remission was confirmed by MRI (**Figures 1F, G**) and undetectable *NPM1::ALK* in CSF, alongside a partial metabolic response on PET/CT (**Figure 1B**). Given the patient’s young age and aggressive disease course, he proceeded to haploidentical allogeneic HSCT in October 2018, two months after initiating alectinib, conditioned with fludarabine, melphalan, and thiotepa. Alectinib was temporarily discontinued on the first day of conditioning but was resumed on day 33 post-transplantation. PET/CT 6 months after alectinib initiation showed a complete metabolic response (**Figure 1C**). He continues alectinib (300 mg twice daily) and has remained in CR for nearly seven years after allogeneic HSCT, with chronic graft-versus-host disease (skin score 2, mouth score 1, eyes score 1) and no alectinib-related toxicity.

**Figure 1:**
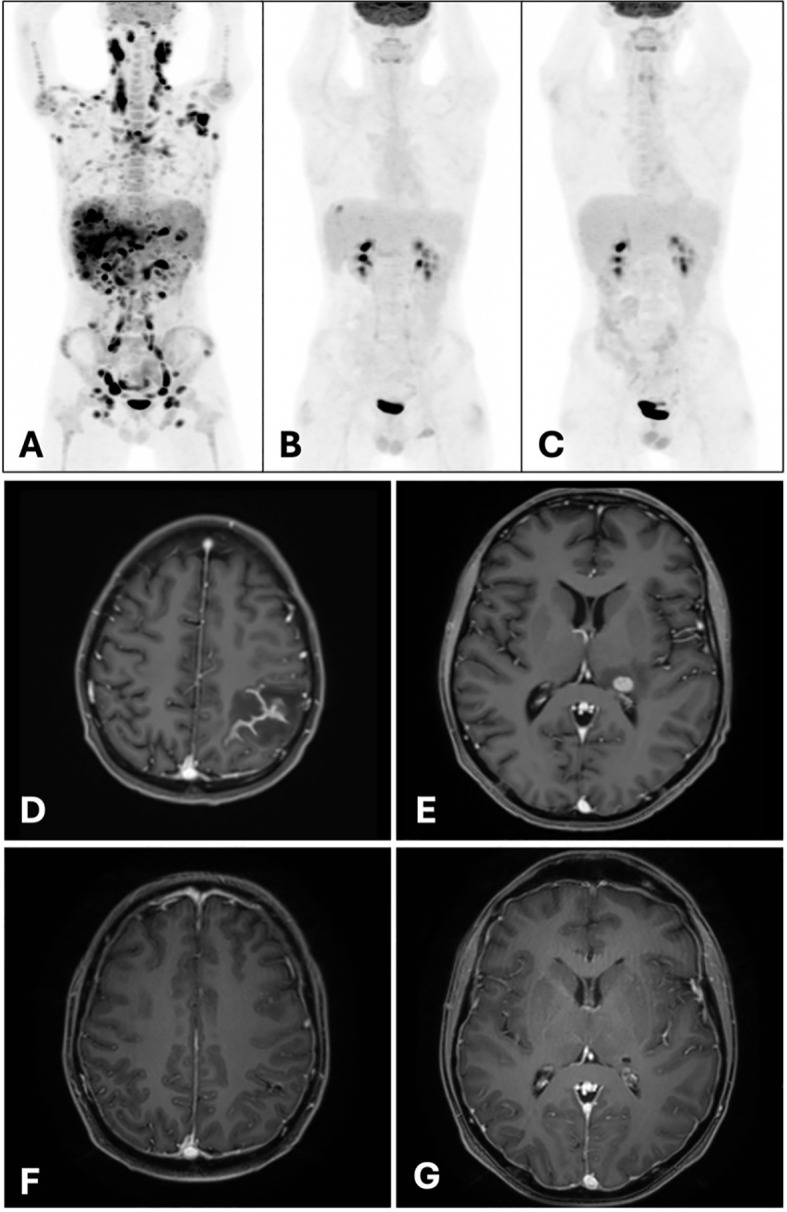
PET/CT and MRI images, Case 1. Maximum intensity projection 18F-fluorodeoxyglucose (18F-FDG) PET images for initial staging revealed hypermetabolic lymphadenopathy disseminated throughout the body and extensive hypermetabolic extranodal lesions, most notably in the liver **(A)**. After treatment with alectinib and crizotinib, interim FDG PET images for response assessment at 3 months show resolution of lymphadenopathy and most of the extranodal lesions, with no new hypermetabolic foci **(B)**. However, several liver lesions remained metabolically active, with residual FDG uptake higher than in the liver parenchyma, indicating an overall Deauville 4 response. Subsequent follow-up FDG PET at 6 months revealed complete resolution of the remaining liver lesions **(C)**. The MRI (T1-weighted axial post-contrast) images show multiple supratentorial parenchymal and leptomeningeal-enhancing lymphomatous lesions with perifocal edema **(D, E)**. Follow-up MRI 3 weeks after initiation of alectinib and intrathecal chemotherapy demonstrates resolution of the lesions with minor residual hemorrhage **(F, G)**.

### Case 2

A 31-year-old female presented with a two-week history of fatigue, vomiting, headache, and neck stiffness in October 2023. Her medical history was notable for ataxia and left hemiparesis following an unspecified meningoencephalitis 17 years earlier. She was initially treated for presumed meningitis with broad-spectrum antibacterial therapy and acyclovir. Within four days, however, her condition deteriorated, with somnolence and muscle weakness progressing to tetraplegia, ophthalmoplegia, bulbar palsy, and hypercapnic respiratory failure, necessitating transfer to the intensive care unit. MRI revealed leptomeningeal enhancement with brain stem predominance (**Figure 2A**). Blood and CSF cultures and a PCR infection panel were negative; MFC and cytology of CSF did not identify a malignant lymphoid population. Due to spinal cord compression, C1 laminectomy with decompression and meningeal biopsy were performed, and a four-day course of methylprednisolone pulse therapy was initiated for suspected CNS sarcoidosis or malignancy. Biopsy confirmed ALK-positive ALCL (**Figures 3A–D**), with NGS RNA-seq detecting inv(2)(p23q35) *ATIC::ALK* fusion gene (Supplementary Material, **Supplementary Figure 1**). Whole body CT scan and bone marrow evaluation revealed no evidence of systemic involvement. The patient received four courses of HD-MTX, with alectinib (600 mg twice daily) introduced after the completion of the first cycle. Within a month, she was weaned from the ventilator, with regression of cranial nerve palsies and right hemiplegia. After 11 weeks of treatment with HD-MTX and alectinib, MRI showed a complete radiological response (**Figure 2B**). Additional treatment involved two cycles of high-dose cytarabine and thiotepa with alectinib, followed by alectinib maintenance (600 mg twice daily). Given the absence of systemic disease and the patient’s poor performance status (ECOG 4) attributable to extensive neurological deficits, HSCT was not pursued. At twenty months after therapy initiation, with ongoing physiotherapy, the patient has demonstrated neurological improvement, with right hemiparesis reducing to grade 3 and left hemiparesis to grade 2. The patient experienced grade 2 constipation during the first month of therapy, considered possibly related to alectinib; no other treatment-related toxicities were noted.

**Figure 2:**
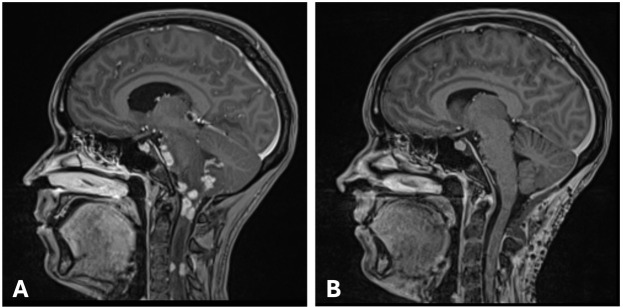
MRI images, Case 2. Baseline MRI (T1-weighted sagittal post-contrast) image shows enhancing nodular lymphomatous lesions involving the third and fourth ventricles, as well as the leptomeninges of the brainstem and cervical spine, with compression of the medulla and occlusion of the fourth ventricle **(A)**. This was initially suspected to be a manifestation of tuberculous leptomeningitis. After cranio-cervical decompression and four cycles of high-dose methotrexate with alectinib, a follow-up MRI image shows the resolution of the lesions **(B)**.

**Figure 3:**
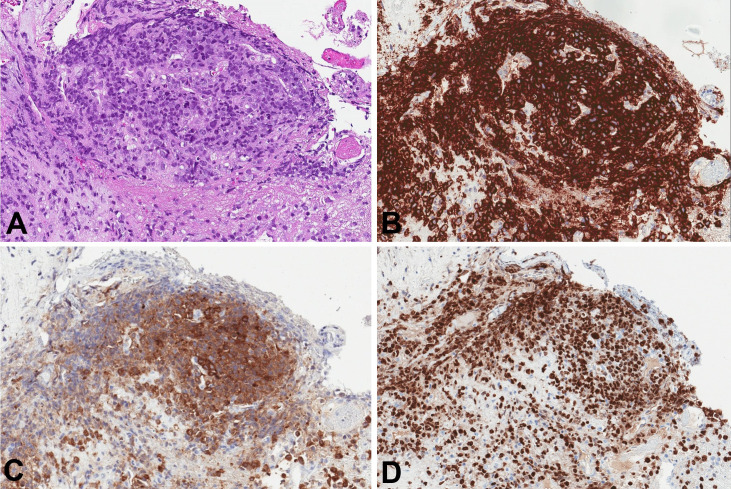
ALK-positive ALCL in the CNS tissue biopsy. The ALCL infiltrate with predominantly perivascular pattern [**(A)**, H&E 100x] demonstrated diffuse expression of CD30 [**(B)**, 100x] and ALK1 [**(C)**, 100x], and the Ki67 proliferation index was up to 95% [**(D)**, 100x].

## Discussion

Several salvage approaches, including second-line combination chemotherapy and BV, followed by autologous or allogeneic HSCT, have been used for refractory or relapsed ALK-positive ALCL (15–18). However, these approaches may not always be feasible in patients with CNS involvement, where the combination of intensive systemic and CNS-directed therapy carries a high cumulative toxicity burden, and patient performance status frequently precludes their use (2, 3, 19).

Case reports suggest that ALK inhibitors, originally developed for non-small cell lung cancer (NSCLC), may be safe and effective in relapsed ALK-positive ALCL (3, 20). The first-generation ALK inhibitor crizotinib demonstrated high efficacy in patients with relapsed or refractory ALK-positive ALCL, with CR rates reaching 80% (3). Nevertheless, as in NSCLC, progression or relapse can occur due to crizotinib-resistance mutations or CNS disease, as crizotinib does not reach therapeutic concentrations in the CSF (3, 14).

Consequently, more ALK-selective second-generation agents with improved CNS penetration, such as alectinib, have been explored in this setting (21, 22). In a study of patients with ALK-positive NSCLC and brain metastases, alectinib achieved a CSF-to-plasma concentration ratio of 86%, confirming its favorable CNS bioavailability (23). A recent phase II trial demonstrated favorable efficacy of alectinib in relapsed or refractory ALK-positive ALCL, with a CR rate of 60% and a 1-year overall survival of 70%, although patients with CNS involvement were not included (24). Alectinib has also proven effective for CNS relapse or progression of ALK-positive ALCL in four case reports and a five-patient series by Rigaud et al. (10–14) Of the nine patients, five had isolated CNS relapses (two following crizotinib monotherapy), two had both systemic and CNS relapses, and two had primary refractory disease. Treatment consisted of alectinib monotherapy in six patients, HD-MTX followed by alectinib in two, and concomitant HD-MTX-based chemotherapy with alectinib in one. All patients achieved CR with rapid clinical responses, with a longest follow-up of 36.5 months (10–14). Furthermore, a recent review of 34 cases of primary isolated CNS ALK-positive ALCL included a 36-year-old woman treated with HD-MTX, followed by alectinib, autologous HSCT, and alectinib maintenance (8). The patient remained in remission for at least 19 months, supporting the efficacy of alectinib combined with chemotherapy in this rare presentation (8).

Experience with other next-generation ALK inhibitors in CNS ALK-positive ALCL is more limited. Lorlatinib, a third-generation ALK inhibitor, exhibits potent blood–brain barrier penetration and has demonstrated efficacy against brain metastases in patients with NSCLC (25). Dolen Burak et al. recently reported a 10-year-old girl with primary CNS ALK-positive ALCL presenting with absence seizures and a left frontal lesion initially mistaken for a cerebral abscess. Following gross-total resection, she received NHL-BFM chemotherapy combined with lorlatinib and focal cranial radiotherapy, achieving complete remission at 18 months of follow-up (26). Brigatinib, another next-generation ALK inhibitor with documented blood–brain barrier penetration, has also been used successfully in this setting. Bertaina et al. described a 13-year-old boy with stage IV ALK-positive ALCL refractory to first-line chemotherapy and BV, who developed CNS progression and achieved CR with combined high-dose chemotherapy and brigatinib, followed by consolidation with allogeneic HSCT and 12 months of brigatinib maintenance, remaining in remission at 24 months post-transplant (27). Brigatinib has also been reported in adult systemic ALK-positive ALCL following BV failure, although CNS-specific data remain limited (28). Dedicated data on these agents in CNS ALK-positive ALCL are still lacking, and further reports are needed to define their role.

In contrast to ALK-positive ALCL, CNS involvement in ALK-negative ALCL and other PTCLs, including PTCL not otherwise specified (PTCL-NOS), carries a markedly worse prognosis (29, 30). In a registry analysis of more than 1,000 patients with PTCL, those with CNS disease at diagnosis had a significantly shorter overall survival than patients without CNS involvement (18.2 vs. 46.0 months), and the optimal treatment remains undefined (29). Across PTCL subtypes, management typically relies on HD-MTX-based regimens combined with intrathecal chemotherapy, which produce only short-lived responses (29–32).

Building on these data, we present two additional cases demonstrating the successful use of alectinib. The first case demonstrates the efficacy of alectinib in a highly aggressive ALK-positive ALCL with CNS involvement. Although intrathecal chemotherapy was administered, its contribution to the overall response remains uncertain given the patient’s prior resistance to multiple chemotherapy regimens. Our second patient benefited from a combination of alectinib and HD-MTX, illustrating the safety and efficacy of this approach. Combining alectinib with CNS-directed chemotherapy may therefore be especially advantageous when a rapid clinical response is needed, as in critically ill patients unfit for multiagent regimens. The role of allogeneic HSCT in patients with relapsed or refractory ALK-positive ALCL with CNS involvement remains controversial. In the previously described studies, two patients underwent consolidation with allogeneic HSCT (13, 14). One patient achieved remission after 2.9 months of alectinib monotherapy and remains in CR 19.1 months post-transplant without alectinib maintenance (14). Conversely, the second patient received 6.5 months of alectinib monotherapy before the transplant, relapsed 30 days post-transplant as skin lymphoma, and responded to the reinitiation of alectinib monotherapy (13). In contrast to these cases, we reinitiated alectinib early after transplant in our first case, primarily due to concern about disease recurrence and the lack of further treatment options. This case represents the longest follow-up reported to date, demonstrating the long-term efficacy and tolerability of alectinib maintenance after allogeneic HSCT. Our two cases reflect an individualized approach: allogeneic HSCT consolidated remission in Case 1 after aggressive systemic and CNS relapse, whereas in Case 2 – with isolated CNS disease and impaired performance status – alectinib maintenance with CNS-directed chemotherapy was selected as a feasible long-term alternative.

The optimal duration of alectinib therapy after achieving complete remission remains unclear, as data on the effects of ALK inhibitor discontinuation are limited. Although some patients may be cured after several years of therapy, immediate relapses following ALK inhibitor discontinuation have been reported, even following prolonged treatment (14, 33). Our cases and those in the literature suggest that alectinib monotherapy is effective and well tolerated for maintaining long-term remissions, with only one reported case of breakthrough relapse of systemic and CNS disease while on therapy (10–14). Indefinite continuation of therapy may therefore be warranted until further data become available.

Our report has several limitations. Most notably, alectinib was administered together with CNS-directed chemotherapy in both cases, and the independent contribution of ALK inhibition therefore cannot be determined. In addition, as a two-patient case series, our observations cannot be generalized. The activity of alectinib monotherapy reported in previous CNS cases^8-^¹ suggests that ALK inhibition contributes meaningfully to the responses observed, but prospective trials are needed to define the optimal treatment combination and the role of each component.

In summary, alectinib, with or without CNS-directed systemic chemotherapy, appears to be an effective treatment approach for both ALK-positive ALCL with CNS relapse and primary isolated CNS disease. Prospective clinical trials should investigate the role of concomitant chemotherapy, the utility of allogeneic HSCT, and the optimal duration of alectinib maintenance.

## Data Availability

The original contributions presented in the study are included in the article/**Supplementary Material**. Further inquiries can be directed to the corresponding author.
