# Supplementary material for: Case Report: Alectinib and CNS-directed therapy for primary or relapsed ALK-positive anaplastic large cell lymphoma with central nervous system involvement
Source: Front Oncol. 2026 Jun 9;16:1679620. doi: 10.3389/fonc.2026.1679620 (PMC13286764; doi:10.3389/fonc.2026.1679620)
Supplement: Supplementary file 1 [file Image1.pdf]

**Supplementary material**

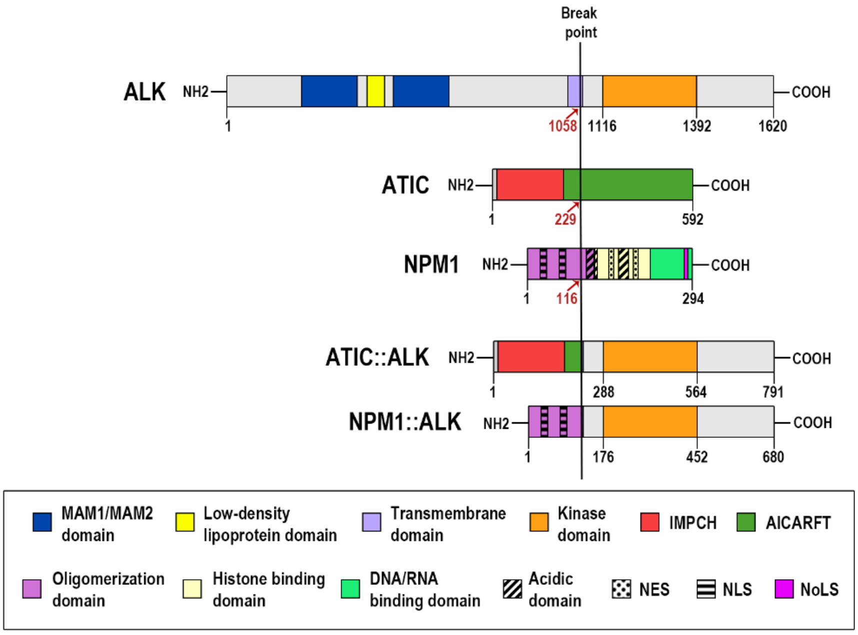


**Supplementary Figure 1. Schematic representation of *NPM1::ALK* and *ATIC::ALK* fusion proteins.** IMPCH, inosine monophosphate cyclohydrolase; AICARFT, 5-aminoimidazole-4-carboxamine ribonucleotide formyltransferase; NES, nuclear export signal; NLS, nuclear localization signal; NoLS, nucleolar localization signal.
